# Civil liability of orthodontists and aligner manufacturers in the treatment with orthodontic aligners

**DOI:** 10.1590/2177-6709.29.1.e24spe1

**Published:** 2024-02-12

**Authors:** Isabela Sales PINHEIRO, Cleverson Raymundo Sbarzi GUEDES, Analina Braga APOLINÁRIO, Fernanda Ramos de FARIA, Sergio Luiz MOTA, Matheus Melo PHITON, Marcio José da Silva CAMPOS, Robert Willer Farinazzo VITRAL

**Affiliations:** 1 Universidade Federal de Juiz de Fora, Faculdade de Odontologia, Programa de Pós-Graduação em Odontologia (Juiz de Fora/MG, Brazil).; 2 Universidade Estadual do Sudoeste da Bahia, Departamento de Saúde (Jequié/BA, Brazil).; 3 Universidade Federal de Juiz de Fora, Faculdade de Odontologia, Departamento de Odontologia Social e Infantil (Juiz de Fora/MG, Brazil).

**Keywords:** Civil liability, Aligners, Invisible aligners, Responsabilidade civil, Alinhadores, Alinhadores invisíveis

## Abstract

**Introduction:**

The use of clear aligners as an alternative to fixed orthodontic appliances has become popular due to the aesthetic demands of adult patients seeking orthodontic treatment. However, orthodontists’ lack of knowledge about the legal consequences of their activities, and the lack of solid scientific evidence raise concerns regarding civil liability in this type of treatment. Marketing campaigns of manufacturing companies often exaggerate promises of results, and ignore the lack of scientific evidence. Patients, as consumers, are protected by the Consumer Protection Code, whereas orthodontists are considered treatment providers. Therefore, they can be held liable for damage caused to patients, whether by subjective or objective fault.

**Objective:**

This article aims to identify the civil responsibilities of orthodontists and aligner manufacturing companies, by means of a literature review, providing basic legal guidance to help professionals protect themselves from possible lawsuits related to treatment with orthodontic aligners.

**Conclusions:**

The study highlights the importance of knowledge of legal notions in treatments with orthodontic aligners by orthodontists, who should legally safeguard themselves through individual written contracts, avoiding obligation of results. In addition, in cases of legal claims, it is possible that the manufacturing companies are jointly and severally liable for possible damages claimed by the patient.

## RESUMO

**Introdução:**

O uso de alinhadores transparentes como alternativa aos aparelhos ortodônticos fixos tem se tornado popular, devido às demandas estéticas dos pacientes adultos em busca de tratamento ortodôntico. No entanto, a falta de conhecimento dos ortodontistas sobre as consequências jurídicas de suas atividades, e a falta de evidências científicas sólidas levantam preocupações em relação à responsabilidade civil nesse tipo de tratamento. Muitas vezes, as campanhas de marketing das empresas fabricantes exageram nas promessas de resultados e desconsideram a falta de evidências científicas. O paciente, como consumidor, é protegido pelo Código de Defesa do Consumidor, e o ortodontista é considerado um fornecedor de tratamento. Portanto, ele pode ser responsabilizado por danos causados ao paciente, seja por culpa subjetiva ou objetiva.

**Objetivo:**

Identificar, por meio de uma revisão bibliográfica, as responsabilidades civis dos ortodontistas e das empresas fabricantes de alinhadores, fornecendo orientações jurídicas básicas para ajudar os profissionais a se protegerem de possíveis demandas judiciais relacionadas ao tratamento com alinhadores ortodônticos.

**Conclusões:**

O estudo destaca a importância do conhecimento de noções jurídicas em tratamentos com alinhadores ortodônticos, devendo o profissional resguardar-se juridicamente por meio de contratos individuais por escrito, prevenindo-se de assumir uma obrigação de resultado com o paciente. Além disso, em casos de demandas judiciais, é possível que as empresas fabricantes respondam solidariamente a possíveis danos reclamados pelo paciente.

## INTRODUCTION

In a scenario in which aesthetic problems are the main reason that lead adult patients to seek orthodontic treatment,^1^ ^,^ ^2^ the so-called “invisible aligners” emerge as a treatment alternative to fixed appliances, allowing orthodontists to offer orthodontic treatment with aesthetic solutions combined with advantages such as increased comfort and easiness of hygiene.^3^ There have been reports of aligners on the market since the 1990s.^4^ The principles proposed by Kesling^5^, associated with intraoral scanning technology, allow for the fabrication of aesthetic and removable aligners.^6^ ^,^ ^7^

Despite the aesthetic appeal being one of the main reasons why patients opt for this type of treatment, it is important for orthodontists to be aware of the patient’s expectations and wishes at the beginning of treatment, avoiding possible dissatisfaction with treatment results, since the number of lawsuits filed against orthodontists has increased considerably in recent years.^8^ ^,^ ^9^ ^,^ ^10^

The patient, as a consumer, can accuse the orthodontist of moral and/or material damage, with the need for civil reparation/compensation in cases where there is dissatisfaction with the orthodontic treatment. Since there is no way of controlling possible complaints from dissatisfied patients, there is no alternative for professionals other than to safeguard themselves by adopting precautions during treatment.^11^ However, orthodontists are usually oblivious of the legal consequences of their activities, and are negligent in relation to both contracts and medical records, either to avoid legal claims or to face them.^11^

## PROPOSITION

The main objective of this study is to identify, based on a literature review, the obligations of orthodontists and aligner manufacturers in relation to their civil responsibilities, in addition to providing basic legal guidance to assist orthodontists on how to safeguard themselves legally in the face of possible lawsuits involving treatment with orthodontic aligners.

## LITERATURE REVIEW

To make the aligners, orthodontists send the intraoral scan and intraoral and extraoral photographs of the patient to manufacturing companies.^7^ Companies then generate a virtual 3D model from the scan and make it available for download and manipulation of dental positions throughout the treatment using specific software. After review and approval by the professional, the aligners are produced and sent to the orthodontist, who delivers them to the patient.^4^ ^,^ ^6^

According to aligner manufacturers, about 20% to 30% of patients treated with aligners may require refinements during the course of treatment.^12^ However, orthodontists report that in fact 70% to 80% of their patients require, during treatment, a change in planning, refinement or the association of fixed appliances with the aligner in order to achieve the desired results.^13^

In addition, some studies indicate that some kinds of tooth movement are more predictable, whereas others are more difficult to perform using aligners.^14^ ^,^ ^15^ ^,^ ^16^ Also, when initial and final setups are superimposed, the final one provided by the company indicates greater corrections, therefore, better results than the real situation presented in the post-treatment models.^14^ ^,^ ^15^ ^,^ ^17^ Therefore, it is important for professionals to consider that the virtual setup is only a graphical representation of the force system, instead of a predictor of the final position of the teeth, thus the desired final position might not be achieved at the end of treatment.^14^ Even with little scientific evidence, the number of companies manufacturing orthodontic aligners has been increasing, with many of them using marketing strategies aimed at the final consumer.^18^ ^,^ ^19^ ^,^ ^20^

According to Shi et al.,^21^ the marketing campaigns displayed on the websites of the manufacturing companies emphasize themes related to aesthetics, comfort, shorter treatment time, better materials, remote monitoring and follow-up applications, and fewer office visits. Out of the 75 brand websites assessed, only 4.5% cited references that confirmed the advertisements. However, these references were predominantly from internal studies conducted by the company itself.

For Artese^22^, the biggest commercial change resulting from the advent of aligners lies in the fact that for the first time an orthodontic product is being advertised directly to the public, and patients have been moved into the category of direct consumers.

The Regional Council of Dentistry of Paraná (CRO-PR) even filed a lawsuit^23^ against a company that manufactures aligners to stop advertising discounts, treatment modalities and comparisons between its products and those provided by other professionals/companies. For this, the CRO-PR was based on the Law n° 6368/76, which states that *“when it comes to drugs, medicines or any other product with the requirement of sale subject to medical or dental prescription, advertising will be restricted to publications that are intended exclusively for distribution to doctors, dental surgeons and pharmacists.”* Despite the granting of the action in favor of CRO-PR, the marketing campaigns continued to be delivered.

The companies even help patients find their nearest accredited professional, through their websites. Another marketing strategy commonly carried out by professionals with the help of manufacturing companies is to invite potential customers to an immersion day in which they are subjected to intraoral scanning, and, through a feature of the scanning software, the patient’s final smile is simulated.^18^ Advertising campaigns lead patients to believe that most malocclusions can be corrected in a shorter time and with the same results, regardless of the dentist who performs the treatment.^19^

The patient is the weaker party in consumer relations, and therefore protected by the Consumer Defense Code (CDC)^24^ ^,^ ^25^ ^,^ ^26^, which defines consumer as any natural or legal person who acquires or uses a product or service. The supplier is any natural or legal person who carries out the activity of production, construction, commercialization and distribution of products or provision of services for remuneration^27^. Thus, the orthodontist is considered a treatment provider to his patient/consumer.^9^ ^,^ ^28^ According to Article 14 of the CDC, the supplier shall be liable for any damage caused to consumers.^9^

Damages to the consumer/patient refer to civil liability, which is characterized as the obligation of the professional towards his patient in order to repair any damage resulting from a voluntary or involuntary action in the exercise of their profession.^28^ ^,^ ^29^ Civil liability can be either subjective, when it is necessary to prove that the alleged damage was due to a faulty action performed by the professional; or objective, when it involves a risk of potential damage to the patient, regardless of whether or not there was fault by the professional.^25^ ^,^ ^27^

In Article 7, the CDC provides for solidarity in the consumer chain. This means that when there is more than one perpetrator of the damage, all will be jointly and severally liable for compensation for the damage provided for in the consumer rules.^30^ Articles 12 and 13 also include the entire chain of suppliers as jointly and severally liable for the product or service they make available on the consumer market,^31^ providing for the possibility of a right of recourse. In other words, the supplier who pays the debt to the consumer will have the right to collect from the other agents responsible for the damage.^26^ It is understood that everyone who contributes to placing a product or service at the mercy of consumers must be liable for possible damage caused to them.^24^

Joint and several liability must be formalized in court by the holder of the injured right - the patient -, and then it will be determined by the ordinary courts.^29^ If the patient has the data of both parties, it is possible that the proceedings already start mentioning the two co-responsible parties. However, when such information is not available at the beginning of the proceedings, it is not a problem to initiate them against only the known debtor, who is most often the professional in charge of treatment. It is possible for both the creditor (the patient) and the debtor (the dentist) to later add a second party (the company supplying the product) to respond to the case, even if it is already underway. In this case, evidence will be needed that the third person included in the file is jointly liable for the debt or obligation. The suppliers, the professional and the manufacturing company, may question the existence of liability, claiming that they have no legal standing as defendants in the action.^30^ ^,^ ^32^

The main benefit of naming more than one person responsible for repairing the damage lies in the fact that, once convicted, it is possible for the defendants to deliberate on how to repair the damage, and the amount of the conviction can be divided between the parties, allowing for security in the fulfillment of the repair of the damage. On the other hand, the risk of using joint and several liability is to increase the number of challenges and arguments contrary to the one presented in the initial application. However, this risk is minimal in view of the greater guarantee of compliance with the obligation, in the enforcement phase.^32^

Joint and several liability has become a huge achievement for Brazilian consumers, enabling all suppliers involved to be held liable for any damage caused to consumers. Therefore, the injured party has no difficulty in identifying the culprit for the damage, avoiding the oppression of the strongest over the weakest in consumer relations.^26^ ^,^ ^31^

## DISCUSSION

Although there is no consensus among literature and jurisprudence on the type of obligation to be assumed by the dental surgeon,^24^ ^,^ ^25^ ^,^ ^29^ most contractual obligations of liberal professionals in Brazil are considered to be of means.^29^ However, some court decisions recognize that the orthodontist has the obligation to achieve the aesthetic and functional result agreed with the patient.^29^

When an orthodontist, with the help of employees of companies that manufacture orthodontic aligners, promotes events with the aim of arousing the patient’s desire for treatment, assuming the possibility of obtaining a smile simulated by a software under a certain value and with a specific quantity of aligners, there is no doubt that both the company and the dental surgeon are assuming an obligation of result with this patient.^33^

In addition, professionals who have the certification required by the company to work with aligners, but are unaware of orthodontic techniques of dental movement because they are not specialists in orthodontics;^19^ or professionals who approve treatments without performing a review of the virtual setup, or even those who guarantee a certain result even knowing the technical and dental movement limitations^14^ ^-^ ^17^ are acting with fault, and therefore falling under subjective liability.

According to Melani and Silva,^34^ 90% of professionals understand civil liability as only giving information to the patient regarding costs, treatment time and objectives. This finding corroborates the study carried out by Guedes^11^, who concluded that orthodontists know little about the legal consequences of their activities, being negligent in relation to contracts, especially due to a failure in the training of these professionals, making it impossible for them to act in a preventive manner.

In the terms and contracts provided by some companies manufacturing orthodontic aligners to practitioners, the companies state that the client (orthodontist) and their practice are fully responsible for treatment planning and use of the products. However, the manufacturing company has a close connection with the professional, promoting it to patients through its websites. In this way, the company places itself as co-responsible for the damages that the patient may have with the treatment. They also state that the software they use presents a computerized approximation of the desired movement for each patient, but the final clinical results may vary. If the company claims that results may be different from what was planned, it contradicts its own marketing campaigns by promising an outcome that may be unattainable.

Despite making such terms and contracts available to dental surgeons, they do not exclude the company from legal claims since, as a supplier of products, it must respond jointly and severally for the repair of damages caused to the consumer, as stated in Article 12 of the CDC.^31^ Joint and several liability will exist when there is any relationship, even if minimal, between the natural/legal person and the right demanded in the legal proceedings, even if there is no direct act performed by all the debtors.

**Figure 1:**
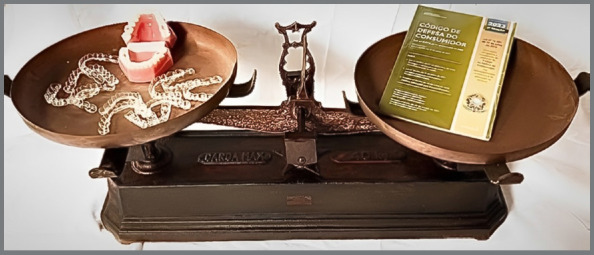
Consumer Protection Code and the practice of orthodontics in treatment with invisible aligners: balance as the main objective.

Article 6 (III) of the CDC states that the consumer’s basic right is to have adequate and clear information about the different products and services, as well as risks presented by them. Therefore, orthodontists who perform treatments with orthodontic aligners should be aware of the limitations of this technique and inform patients of the possibility of using auxiliary devices in the final stages of treatment to obtain better results, such as the installation of fixed appliances, use of elastics or mini-implants^35^ ^,^ ^36^ (Fig 1). 

From this review, it can be noted that, although the manufacturing companies provide standardized contracts to professionals, stating that they are not responsible for any damage resulting from the treatment, according to the CDC, they have as much responsibility as the professional, because they are suppliers of the product. In addition, through their advertising campaigns aimed at end consumers, they commit themselves to an obligation of result and may therefore be jointly and severally liable in cases of legal claims. 

## CONCLUSIONS

Therefore, it is possible to conclude that:

» The professional must know the patient’s expectations, and clarify the treatment that will be carried out, in order to avoid possible dissatisfaction.
» In cases in which the virtual setup is presented to the patient, the professional should explain that it is a graphical representation of dental movement, and that it may not be faithfully reproducible.
» The patient should be aware that new aligners may be required or auxiliary mechanics may be used to approximate the desired result.
» All information passed on to the patient should be formally recorded in an individual contract, so that the practitioner is protected from assuming an outcome liability with the patient.
» The contract must contain all the information relevant to the treatment to be performed, including treatment limitations and possibilities of failure, as they are an instrument of defense for the orthodontist in the event of a lawsuit.
» In the event of patient dissatisfaction and a possible lawsuit, the accused orthodontist or even the patient themselves may request that the aligner companies respond jointly and severally to the possible damages to the patient, since joint and several liability exists when there is any relationship between the natural/legal person and the right demanded in the lawsuit, even if there is no direct act performed by all debtors.
